# The Apparent Asymmetrical Relationship Between Small Bowel Bacterial Overgrowth, Endotoxemia, and Liver Steatosis and Fibrosis in Cirrhotic and Non-Cirrhotic Patients: A Single-Center Pilot Study

**DOI:** 10.3389/fmed.2022.872428

**Published:** 2022-04-26

**Authors:** E. Scarpellini, L. Abenavoli, V. Cassano, E. Rinninella, M. Sorge, F. Capretti, C. Rasetti, G. Svegliati Baroni, F. Luzza, P. Santori, A. Sciacqua

**Affiliations:** ^1^Hepatology and Internal Medicine Unit, “Madonna del Soccorso” General Hospital, San Benedetto del Tronto, Italy; ^2^T.A.R.G.I.D., Gasthuisberg University Hospital, KULeuven, Lueven, Belgium; ^3^Department of Health Sciences, University “Magna Græcia”, Catanzaro, Italy; ^4^Department of Medical and Surgical Sciences, University “Magna Græcia”, Catanzaro, Italy; ^5^Clinical Nutrition Unit, Department of Medical and Surgical Sciences, Fondazione Policlinico A. Gemelli IRCCS, Rome, Italy; ^6^Department of Translational Medicine and Surgery, Università Cattolica del Sacro Cuore, Rome, Italy; ^7^Gastroenterology and Endoscopy Unit “Madonna del Soccorso” General Hospital, San Benedetto del Tronto, Italy; ^8^Gastroenterology Clinic, “Riuniti University Hospital”, Polytechnics University of Marche, Ancona, Italy

**Keywords:** gut microbiota, dysbiosis, small intestinal bacterial overgrowth, liver steatosis, fibrosis

## Abstract

**Introduction:**

Gut microbiota are a complex ecosystem harboring our intestine. They maintain human body equilibrium, while their derangement, namely, “dysbiosis“, has been associated with several gastrointestinal diseases, such as liver steatosis (NAFLD) and liver cirrhosis. Small intestinal bacterial overgrowth (SIBO) is an example of dysbiosis of the upper gastrointestinal (GI) tract.

**Aim:**

The aim of this study is to evaluate the relationship between SIBO and levels of endotoxemia and grade of liver steatosis (LS) and liver fibrosis (LF) in hepatologic patients.

**Materials and Methods:**

Consecutive outpatients referred to our hepatology clinic were tested for SIBO by the lactulose breath test (LBT) and peripheral blood levels of endotoxemia; LS grading and LF were assessed by abdominal ultrasound and transient elastography, respectively.

**Results:**

Fifty-two consecutive patients (17 with alcohol abuse (4.5 ± 0.8 alcohol units per day), 4 with HCV and 2 with HBV infection, 24 of metabolic origin, 2 of autoimmune origin, and 3 with cholangiopathies; mean age 54.7 ± 8.3 years, 31 F, BMI 24.1 ± 1.1 Kg/m^2^) and 14 healthy volunteers (HV) (mean age 50.1 ± 4.3 years, 9 F, BMI 23.3 ± 1.1 Kg/m^2^) were enrolled. SIBO prevalence was significantly higher in cirrhotic (LC) vs. non-cirrhotic (LNC) patients and vs. HV (all, *p* < 0.05), with a significant positive trend according to Child-Pugh status (all, *p* < 0.05). SIBO prevalence was not correlated with LS stages (all, *p* = NS). Consensually, endotoxin levels were significantly higher in LC vs. LNC and vs. HV (all, *p* < 0.05) and significantly correlated with LF in patients with LC, according to Child-Pugh status (all, *p* < 0.05).

**Conclusion:**

This study shows that SIBO prevalence and relative endotoxin blood levels seem to be significantly associated with the grade of LF vs. LS in LC. SIBO is also present under pre-cirrhotic conditions, but its prevalence seems to correlate with liver disease irreversible derangement.

## Introduction

Liver steatosis (namely, nonalcoholic fatty liver disease, NAFLD) is a hallmark of fat deposition in the liver. This is the first step of a complex and sometimes progressive process that can lead to liver cirrhosis (LC) and hepatocellular carcinoma (HCC) (1). The main stages of NAFLD are nonalcoholic steatohepatitis (NASH) with ballooning of fat deposits, fibrosis until cirrhosis, and HCC (2). The histopathology of NAFLD is very similar to those of alcoholic liver disease (ALD). However, NAFLD diagnosis requires the exclusion of a daily alcohol intake of more than 30 g for men and 20 g for women (3). It is worth mentioning a new pathophysiological entity, recently introduced in hepatology. It falls in between NAFLD and ALD, according to the synergy of a lower alcoholic intake than ALD, and the consensual effect of a high-fat diet, namely, “metabolic-associated fatty liver disease” (MAFLD) (4, 5). MAFLD is also characterized by hepatic steatosis in patients with three metabolic conditions: obesity/overweight, diabetes, and metabolic dysregulation, either alone or in combination (5). It is interesting to note that MAFLD seems to have faster and more severe progression of hepatic fibrosis vs. both ALD and NAFLD (6).

Nowadays, NAFLD is considered the leading cause of LC and HCC because of consensual reduction of viral hepatitis incidence (7). Lifestyle changes, dietary interventions, and regular physical activity are the mainstay for steatosis reversal and liver fibrosis prevention (8).

An emerging physiopathological actor in NAFLD pathogenesis is gut microbiota. Indeed, the liver is directly exposed to intestinal-derived antigens (e.g., Gram-negative lipopolysaccharides, LPSs) through portal circulation (9, 10). These pathogen-associated molecular patterns (PAMPs) can trigger an inflammatory cascade within the liver leading to NASH. In this vicious circle (called “gut-liver axis”), loss of microbial diversity and abundance, namely, “dysbiosis”, seems to be a crucial factor for the beginning and progression of NAFLD (11).

Therefore, gut dysbiosis can affect both hepatic lipid metabolism disruption and inflammatory processes through altered intestinal permeability (9). The gut microbiota of patients with NAFLD patient show increased concentration of Bacteroidetes and reduction of Firmicutes vs. healthy subjects, with significant reduction in short-chain fatty acid-producing families and genera (12). However, there are controversial pieces of evidence correlating the increased abundance of one or several specific bacterial strains with NAFLD in its progressive stages (13).

Small bowel bacterial overgrowth (SIBO) is a clinical syndrome characterized by the outnumber of bacteria in the small intestine exceeding 10^6^ CFU per ml of jejunal aspirate (14). SIBO association with NAFLD is a controversial emerging fact in the literature. On the other hand, its presence in advanced liver disease stages, such as liver cirrhosis and HCC, is well known (15). In detail, several systematic reviews of literature and meta-analyses have confirmed a statistical association between SIBO and NAFLD stages (16, 17). However, recent original research studies by Guimaraes and Nier have shown how the pathophysiological link between SIBO and NAFLD stages is far from being explained (18, 19). Indeed, another index of gut dysbiosis, namely, peripheral blood endotoxemia (one of the most common PAMPs), seems to be involved in the passage from NAFLD to the NASH stage of liver disease (18, 19).

In this single secondary-center prospective comparative study, we aimed to evaluate the prevalence of SIBO and levels of endotoxemia as an index of LPS concentrations in consecutive cirrhotic and non-cirrhotic patients admitted to our hepatology outpatient clinic.

## Materials and Methods

### Study Design

From January to July 2018, patients admitted to our hepatology outpatient clinic were consecutively enrolled with informed consent. The patients underwent complete clinical evaluation, abdominal ultrasound, transient elastography, endotoxin peripheral blood dosage, and lactulose breath test (LBT) for SIBO assessment on separate consecutive days.

The study was approved and registered by ASUR Marche Regional Ethical Committee.

### Inclusion and Exclusion Criteria

With informed consent, hepatology outpatients aged 18–65 years were admitted to the study. Patients with NAFLD, ALD, NASH, ASH, autoimmune hepatitis (AIH), cholangiopaties (primary biliary cholagiopathies, sclerosing cholangitis), HBV, or HCV were included in the study.

NAFLD diagnosis was made according to the detection of liver steatosis with abdominal ultrasound and liver stiffness (LF) values ranging between F0 and F1 at transient elastography. Furthermore, daily alcohol intake of more than 30 g for men and 20 g for women was excluded (3). NASH diagnosis was made according to LF values, assessed with transient elastography, ranging between F2 and F3 (3).

ALD diagnosis was made according to detection of liver steatosis by abdominal ultrasound, with LF values ranging between F0 and F1 at transient elastography. A daily alcohol intake of more than 30 g for men and 20 g for women was required (3). ASH diagnosis was made according to LF values, assessed with transient elastography, ranging between F2 and F3 (3).

Autoimmune origin or cholangiopathy diagnosis was accepted after autoimmune antibody detection and confirmation by liver biopsy (20).

Liver cirrhosis, characterized by tissue fibrosis and the presence of regenerative nodules replacing functioning hepatic tissue, is presented with increased portal pressure with splanchnic vasodilation and blood flow (21). Thus, clinical LC diagnosis was made by the presence of splenomegaly, anemia, hypoalbuminemia, thrombocytopenia, and ascites. Liver stiffness, confirming the clinical and histological diagnosis, was assessed by transient elastography (cutoff value for LC of any cause: > 16 kPa). Liver cirrhosis stage classification followed the Child-Pugh score. Gravity and mortality risk of patients with LC were assessed using the MELD score (21).

Hepatic encephalopathy (HE) was assessed by the presence and grading of the following neurologic symptoms and signs: In grade 0, patients present with just mild changes in memory, concentration capability, coordination, and no asterixis. The latter, together with altered orientation, altered sleep (hypersomnia/insomnia), slowed ability to perform mental tasks (diagnosed and quantified by neuropsychological tests), decreased attention, mood alterations (ranging from depression to euphoria) are typical of grade I. In grade II, lethargy and/or apathy is present; mild to moderate disorientation, personality change with increased irritability, slurred speech can also be apparent. Severe confusion with somnolence, reversible by verbal stimuli, gross disorientation, speech incoherence are signs of the third grade of HE. In the fourth grade, there is a coma (21).

Uses of lactulose, proton pump inhibitors (PPIs), and beta-blockers were recorded.

Exclusion criteria were: use of pre- and probiotics, antibiotics, prokinetics, and any other medication potentially altering gut microbiota in the 30 days prior to enrolment; presence of cancer disease; history of abdominal surgery; presence of chronic renal failure, thyroid, respiratory, or cardiac disease and failure.

### Patient Evaluation

Anthropometric measurements, including body mass index (BMI), and complete physical examination, were performed.

Peripheral blood samples for hepatic function assessment (transaminases, G-GT, alkaline phosphatase, bilirubin, albumin, complete blood count, coagulation tests, standard urine test, ammonia) were collected after 12-h fasting.

In particular, ammonia measurements were collected with EDTA tubes on venous samples. After immediate transport on ice to the laboratory, the samples were spun at 1,885 g for 10 min. Plasma ammonia concentration was measured on a Modular P800 analyzer (Roche, Roche Diagnostics, United States) using an enzymatic kinetic method with a final photometric measurement of produced NADPH at 340 nm (measuring range: 5.87–587 lmol/L; reference values from 11 to 50 lmol/L for women and 15–55 lmol/L for men, and intra- and interassay CV ≤ 8%) (22).

### Abdominal Ultrasound

Abdominal ultrasound evaluation was undertaken after the patient overnight fasting.

Qualitative LS grading was classified as mild, moderate or severe, or grade 0–3 with 0 being normal (23, 24). Grade 1 (namely, mild) consisted of mild diffuse increase in fine echoes in the hepatic parenchyma with normal visualization of the diaphragm and intrahepatic vessel borders; grade 2 (namely, moderate) consisted of a moderate diffuse increase in fine echoes with slightly impaired visualization of intrahepatic vessels and the diaphragm; grade 3 (namely, marked) consisted of marked increase in fine echoes with poor or no visualization of intrahepatic vessel borders, the diaphragm and posterior portion of the right lobe of the liver (25).

The presence of portal hypertension was assessed; values of portal velocity <13 cm/s were considered as cutoff for portal hypertension diagnosis (26).

### Transient Elastography

Transient elastography (TE), first developed as Fibroscan^®^ (Echosens, Paris, France), consists of a vibrator that generates low-frequency shear waves through the liver, which are then transmitted to an ultrasound receiver. The velocity of the waves is dependent on tissue elasticity; therefore, the rate of propagation through the liver can be used as a measure of liver stiffness and converted into a numerical value (kPa) (27, 28). TE was performed using the “Fibroscan touch" model and with patient lying in dorsal decubitus position and with the right arm in maximal abduction, using the right lobe of the liver through the intercostal space at the site of biopsy. All measurements were started using the M probe and transitioned to the XL probe only if the initial measurement was “invalid”, as guided by the equipment software.

A ten-millimeter diameter core of tissue with a depth between 20 and 40 mm from skin surface was measured. Ten shots within 2–3 min were performed (at least 60% valid shots). The median liver stiffness measured by shots represents liver stiffness median (LSM). The ratio of the interquartile range (range in which 50% of all shots fall, IQR) to liver stiffness measurement <0.3 was taken as valid results (29).

Cutoffs used for liver stiffness, according to liver disease causative diagnosis, were (26):

- patients with HCV: F0-F1 < 7.6 kPa; F2 7.6–10.9 kPa; F3 10.9–15.3 kPa; F4 > 15.3 kPa,
- patients with HBV, AIH, or cholagiopathies: F0-F1 < 7 kPa; F2 7–8.2 kPa; F38.2–11.3 kPa, F4 > 11.3 kPa, and
- patients with NAFLD or ALD: F0–F1 < 6.6 kPa; F2 6.6–7.8 kPa; F3 7.8–10.4 kPa; F4 > 10.4 kPa.

### Endotoxin Level Dosage

Serum endotoxin measurement was performed by a chromogenic Limulus Amebocyte Lysate (LAL) assay with a QCL-1000™ LAL Endpoint Assay (Lonza, NJ, United States) commercial analysis kit on sera previously stored at −70°C (18).

### Lactulose Breath Test

Diagnosis of SIBO was established by hydrogen (H_2_) LBT. On a dedicated day, all the patients underwent LBT under standard conditions (30). The patients should have not received laxatives in the 30 days preceding the test. They were asked to have a carbohydrate-restricted dinner on the day before the test and to fast for at least 12 h to minimize basal H2 excretion. Physical exercise was not allowed for 30 min before and during the test. End-alveolar breath samples were collected immediately before lactulose ingestion. Then, a dose of 10 g of lactulose in a 20-ml solution was administered, and samples were taken every 15 min for 4 h using a two-bag system. The two-bag system is a device consisting of a mouthpiece, a *T*-valve, and two collapsible bags (the first one collects dead space air, and the second one collects alveolar air). The breath samples were aspirated from the bag into a plastic syringe. The samples were analyzed immediately for H2 using a model DP Quintron Gas Chromatograph (Quintron Instrument Company, Milwaukee, WI, United States). The results were expressed in parts per million (ppm). A normal LBT was defined as the absence of an early rise in H2 excretion of >20 ppm within the first 90 min (30).

### Statistical Analysis

Preliminarily, sample size calculation was operated to detect 30% differences between groups.

In this single secondary-center prospective comparative study, a statistical analysis of the data collected was performed with the Instat^®^ program. According to their normal or not normal distribution, data are presented as mean or median ± SD.

Comparisons of endotoxin levels, SIBO prevalence, antropometric and laboratorial biochemical parameters between groups of patients were conducted by Mann-Withney, Kruskal-Wallis, and Fisher exact tests when needed. Pearson regression coefficient was calculated to evaluate the correlation between serum endotoxin levels, SIBO prevalence and laboratorial biochemical parameters, and Child-Pugh and MELD scores. Significant difference was considered at the 5% level, namely, *p* < 0.05 (31).

## Results

In the present study were enrolled: 52 patients [mean age 54.7 ± 8.3 years, 31 F, BMI 24.1 ± 1.1 Kg/m^2^; 17 with alcohol abuse (4.5 ± 0.8 alcohol units per day), 4 with HCV and 2 with HBV infection, 24 of metabolic origin, 2 of autoimmune origin, and 3 with cholangiopathies] and 14 healthy volunteers (mean age 50.1 ± 4.3 years, 9 F, BMI 23.3 ± 1.1 Kg/m^2^).

Twenty-two patients had liver cirrhosis diagnosis (10 with alcohol abuse, 2 with HCV- and 1 with HBV-infection, 8 of metabolic origin, 1 with cholangiopathies) and 30 did not (7 with alcoholic abuse, 2 with HCV- and 1 with HBV-infection, 16 of metabolic origin, 2 of autoimmune origin and 2 with cholangiopathies).

There was no statistical difference in anthropometric parameters between the cirrhotic and non-cirrhotic patients except for autoimmune liver diseases and cholangiopathy prevalence (Table 1).

**Table 1:** Anthropometric (body weight and BMI) and non-anthropometric data of the enrolled patients.

**Patients characteristics**	**Cirrhotic (*n =* 22)**	**Non-cirrhotic (*n =* 30)**	***p*** **-value**
Mean age	55.1 ± 1.2 years	52.1 ± 1.1 years	NS
Female sex	*n =* 14	*n =* 17	NS
BMI	22.3 ± 1.3 Kg/m^2^	24.8 ± 1.4 Kg/m^2^	NS
Alcohol abuse (ALD, ASH)	*n =* 10(5.1 ± 1.0 AU)	*n =* 7(4.8 ± 0.9 AU)	NS
HCV and HBV infection	*n =* 2; 1	*n =* 2; 1	NS
Metabolic origin (NAFLD, NASH)	*n =* 8	*n =* 16	NS
Autoimmune/ Cholangiopathies	*n =* 0/1	*n =* 2/2	0.05
LS grade 1, 2, 3	N/A	*n =* 7; 15; 8	N/A
LF (F0-F1; F2-F3)	18.1 ± 0.7 kPa (F4)	*n =* 12 (4.4 ± 0.5 kPa) (F0-F1); *n =* 18 (10.8 ± 0.4 kPa) (F2-F3)	N/A

*NS, not significant; BMI, body mass index; ALD, alcoholic liver disease; AU, alcohol unit per day; NAFLD, non-alcoholic fatty liver disease; LS, liver steatosis; N/A, not applicable; LF, liver fibrosis (assessed by transient elastography)*.

Eighteen patients with LC had signs of portal hypertension (mean portal velocity of 9.3 ± 0.8 cm/s; all of them with detection of esophageal varices and congestive gastropathy), and five of them had previously undertaken procedures for variceal bleeding. Fifteen out of the 18 cirrhotic patients with signs of portal hypertension were under beta-blocker treatment for bleeding control. Six patients with LC were classified as Child A, 10 as Child B, and 6 as Child C, according to the Child-Pugh score.

In the non-cirrhotic patients' group, LS had the following distribution: 7 had mild, 15 moderate, and 8 severe LS. Alcohol abuse and NAFLD had LS ranging from moderate to severe in 18 patients (Table 1).

In the non-cirrhotic group of patients, LF was distributed as follows: 12 with F0-F1 (mean value 4.4 ± 0.5 kPa) and 18 with F2-3 (mean value 10.8 ± 0.4 kPa). The mean value of stiffness in the cirrhotic group of patients was 18.1 ± 0.7 kPa (namely, F4) (Table 1).

**Figure 1:**
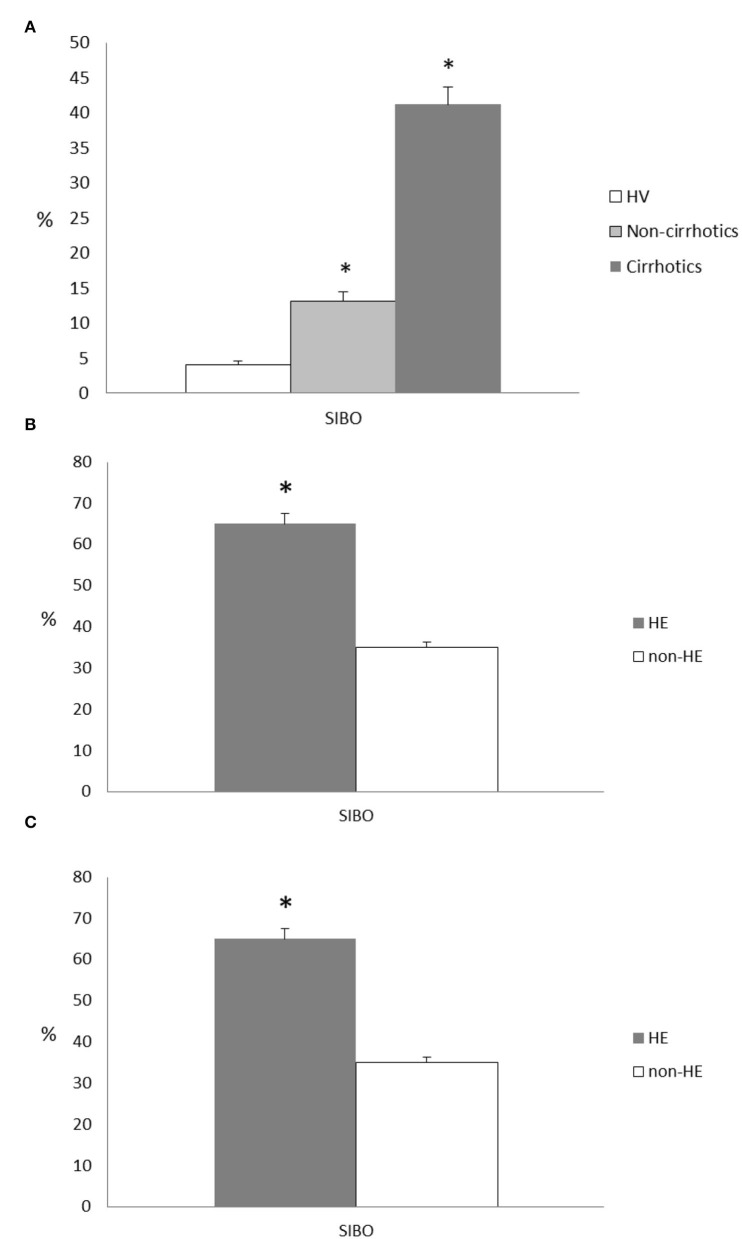
**(A)** SIBO prevalence in the healthy volunteers (HVs) vs. the cirrhotic and non-cirrhotic patients; all *p < 0.05. SIBO, small intestinal bacterial overgrowth. **(B)** SIBO prevalence according to Child-Pugh status in the cirrhotic patients; *p < 0.05. Comparisons between Child-Pugh class B or C and class A were made by Fisher's exact test. SIBO, small intestinal bacterial overgrowth. **(C)** SIBO prevalence according to presence or absence of HE in cirrhotic patients; *p < 0.05. SIBO, small intestinal bacterial overgrowth; HE, hepatic encephalopathy.

SIBO prevalence was significantly higher in cirrhotic vs. non-cirrhotic patients (41.2 ± 2.5 vs. 13.1 ± 1.4%, *p* < 0.05) (Figure 1A). Moreover, both the cirrhotic and non-cirrhotic patients had a higher SIBO prevalence than the healthy controls (SIBO prevalence of 4.1 ± 0.5%; *p* < 0.05). Furthermore, in the cirrhotic patients, SIBO prevalence was increased according to increasing Child status (all, *p* < 0.05) (Figure 1B) and MELD score (all, *p* < 0.05). Interestingly, SIBO prevalence was significantly correlated with hepatic encephalopathy (HE) presence (*p* < 0.05) (Figure 1C) and relative values of ammoniemia (95 ± 1.8 vs. 45 ± 1.2 lmol/L, cirrhotic patients with and without HE presence, respectively, *p* < 0.05). SIBO prevalence was not significantly higher in patients LC with portal hypertension vs. those without portal, because of the small number of patients without portal hypertension.

SIBO prevalence was not correlated with LS grading in the non-cirrhotic group of patients (*p* = NS).

In particular, we did not find a significant difference in SIBO prevalence according to growing LS grading in these patients (mild vs. moderate and severe, both *p* = NS). Moreover, we did not observe a difference in terms of SIBO prevalence according to LF grading under non-cirrhotic conditions (NAFLD vs. NASH and ALD vs. ASH, both *p* = NS).

**Figure 2:**
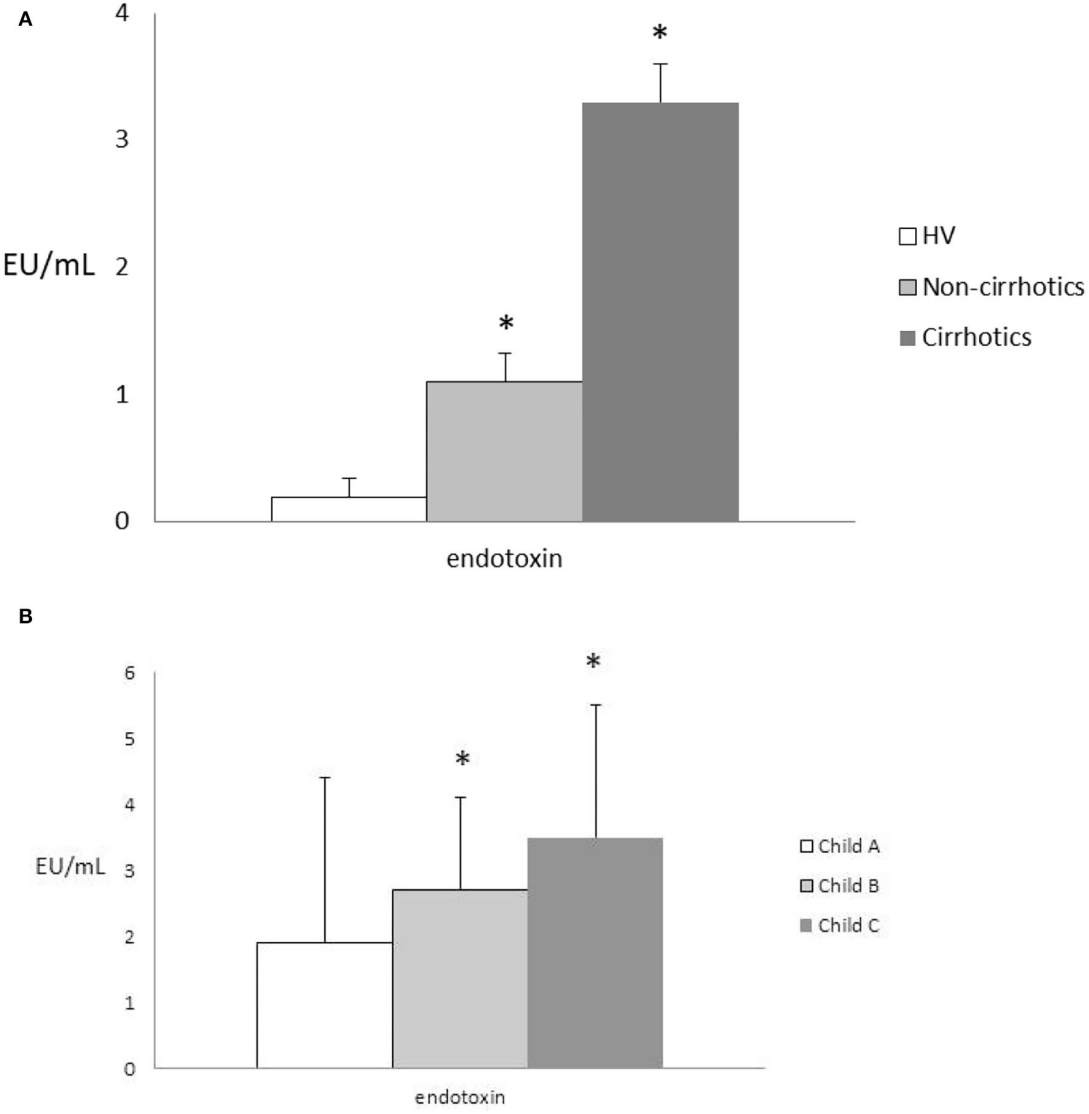
**(A)** Endotoxin levels in the HVs vs. the cirrhotic and non-cirrhotic patients; all *p < 0.05. HVs, healthy volunteers. **(B)** Endotoxin levels according to Child-Pugh status in the cirrhotic patients; all, *p < 0.05. Comparisons between endotoxin values were made by the Kruskal-Wallis test and the *post-hoc* Dunn test.

Endotoxin blood levels were significantly higher in the cirrhotic vs. the non-cirrhotic patients and vs. the healthy controls (3.3 ± 0.23 vs. 1.1 ± 0.3 and.2 ± 0.15 EU/ml, all *p* < 0.05) (Figure 2A). Its levels were correlated with the fibrosis stage in cirrhotic patients, according to growing Child-Pugh status (all, *p* < 0.05) (Figure 2B) and MELD score (all, *p* < 0.05). In the cirrhotic patients, presence of HE and relative ammonia levels were significantly associated with higher endotoxin levels (*p* < 0.05).

In the non-cirrhotic group of patients, endotoxin blood levels did not correlate with LS grading (r = NS). Moreover, we did not observe a difference in terms of endotoxin levels according to LF grading under non-cirrhotic conditions (NAFLD vs. NASH and ALD vs. ASH, both *p* = NS).

Interestingly, the cirrhotic patients with SIBO did not show significantly higher endotoxin values vs. those without SIBO (*p* = NS) (Table 2). The same result was observed in the non-cirrhotic group of patients (*p* = NS) (Table 2).

**Table 2:** Comparison of endotoxin values among cirrhotic patients with and without SIBO and among non-cirrhotic patients with and without SIBO.

**SIBO prevalence**	**Endotoxin value in Cirrhotics (*n =* 22)**	**Endotoxin value in non-cirrhotics (*n =* 30)**	***p*** **-value**
SIBO +	3.1 ± 0.23 EU/mL	2.2 ± 1.1 EU/mL	NS
SIBO –	2.9 ± 0.20 EU/mL	1.3 ± 0.20 EU/mL	0.05

*SIBO, small bowel bacterial overgrowth; NS, not significant*.

## Discussion

This study showed a significant increase in SIBO prevalence in the cirrhotic and non-cirrhotic patients (with hepatopathy) vs. the healthy population. In the cirrhotic patients, SIBO prevalence increases along with Child-Pugh class and MELD score. The measure of a peripheral product of deranged gut microbiota, namely, blood endotoxemia, followed this trend accordingly. However, endotoxemia did not increase with LS grading.

Several scientific reports showed an association between gut microbiota derangement and liver diseases derived from viral hepatitis, cholangiopathies, autoimmunity, alcohol abuse, and, more recently, altered metabolism (13, 32–35). Since the prevalence of liver diseases of viral origin (especially HCV infection) has significantly dropped because of the use of direct-acting antivirals worldwide (35), we focused our attention mainly on metabolic and/or alcoholic causes of liver disease (NAFLD and ALD). We confirmed that SIBO is significantly associated with liver disease. This finding is in agreement with previous reports from the literature (32–36). However, SIBO prevalence in NAFLD is significantly variable, ranging between 17, 26.2, and 60% according to the reports (32–37). More recently, Guimaraes et al. described SIBO prevalence in a population of patients with NAFLD to be of 26.2 % (18). However, in this study, only patients with NAFLD and NASH were studied compared to our investigation.

We confirmed the association between SIBO and NAFLD, but we did not notice a difference in stages of pre-cirrhotic conditions, such as NAFLD and NASH or ALD and ASH. This finding would suggest that the intermediate stage of liver disease, either of metabolic or alcoholic abuse origin, does not seem to impact or to be affected by deranged upper GI tract dysbiosis, namely, SIBO. This finding is in contrast with other reports in the literature that suggest bigger prevalence of SIBO in NASH vs. NAFLD, or at least its evolving histologic stage of ballooning (17, 18). In the present study there is lack of histopathology data, a more direct measure of LF than fibroscan. This may explain differences with the study by Guimaraes et al. (18), from metanalysis of data performed by Wijarnpreecha et al. (17) and reviewed by Goshal et al. (16).

Another possible explanation of this result is that SIBO is a clinical syndrome with symptoms common in other malabsorptive conditions, such as irritable bowel syndrome (IBS) (38). Thus, SIBO does not specifically describe the entire dysbiosis of the whole GI tract belonging to hepatologic patients. Moreover, SIBO does not represent the different expressions of dysbiosis according to different liver diseases (cirrhotic and non-cirrhotic conditions), perhaps described in detail in the literature (39, 40). In fact, colonic bacterial flora, described more accurately by the fecal microbiota study, has also been described under cirrhotic and non-cirrhotic conditions to be altered (41–43).

Blood endotoxin concentrations are consensual to the findings of SIBO, and these data are also in agreement with literature reports describing increased LPS levels in NAFLD, NASH, ALD, ASH, and liver cirrhosis (44). Indeed, we can explain increasing endotoxin levels according to Child-Pugh status and MELD score as a consequence of worsening portal hypertension in patients with liver cirrhosis. This was not affected by the use of beta-blockers in our investigation, probably because of the large use of them by the studied LC population. Furthermore, increasing endotoxin levels in cirrhotic patients according to a more deranged disease stage can also be a result of increased prevalence of SIBO because of proton pump inhibitor major use and slowed GI transit, typical of cirrhotic patients (45). Thus, the increased SIBO prevalence and levels of endotoxin according to worsening Child status and worsening MELD-related mortality risk of cirrhotic patients recorded in this study seem to have a bi-directional association that deserves further investigation.

LS and its progression are not correlated with SIBO prevalence and endotoxin concentrations. These data are not in agreement with the literature showing a significant association between SIBO and NAFLD and its LS grades (37). Indeed, a recent report by Nier et al. showed that endotoxin plasma levels and markers of inflammation were significantly higher in NAFLD compared to controls and increased with the severity of hepatic steatosis (19). These discrepancies can be explained by both the small sample size and the absence of a significant portion of obese patients with NAFLD among the higher classes of LS in this study. In fact, obesity has a major weight on fat deposition severity in patients with NAFLD (46). Another reason explaining the difference between this and other reports is the absence of histopathological findings: “hepatocyte ballooning” seems to be the liver alteration most associated with SIBO (18).

We observed higher endotoxin levels vs. healthy people; this result confirms reports from the literature showing increased endotoxin concentrations in starting metabolic- and alcohol-related liver conditions (47). However, there are no cutoff values for serum endotoxin levels in patients with NAFLD/ALD. Indeed, these patients are shown to be also hyper-responsive to low levels of endotoxin (48, 49). Finally, because of the study being a pilot version and small sample size, we were not able to perform a preliminary multivariate model analysis for endotoxin levels (50).

It is interesting to note how SIBO prevalence in both the cirrhotic and non-cirrhotic patients did not affect the values of endotoxin. This fact reinforces the hypothesis that SIBO does not represent a valuable surrogate marker of dysbiosis in liver diseases, and that endotoxin only describes the peripheral product of Gram-negative dysbiosis (51). These data are in partial agreement with some reports from the literature and, on the other hand, differ from some others that show a significant difference between patients with NAFLD and those with NASH (52, 53). The difference between this and other studies may also arise from the small sample size of patients with NAFLD/ALD of this exploratory single-center study. Thus, it is not possible to have a sufficient number of patients belonging to NALFD and NASH groups to be compared. However, the main aim of this study was to evaluate SIBO prevalence and endotoxin levels in a population of consecutive hepatologic outpatients either cirrhotic or non-cirrhotic.

This study has several limitations: first, the small sample size from a single secondary center has conditioned the non-homogeneity of the study groups. Although alcohol and disorders of metabolism (namely, NAFLD) are most represented in both the cirrhotic and non-cirrhotic groups, these numbers are small and may bias result significance. On the other hand, alcohol and dysmetabolism may have a major weight on the evaluation of the results' significance. Furthermore, the perspective and exploratory nature of this study can also explain the heterogeneity of the groups studied. Second, the LBT has limited sensitivity and specificity in detecting SIBO (40 and 80%, respectively) in clinical practice vs. glucose breath test and the suggested gold standard, namely, jejunal aspirate (30). Some reviews and meta-analyses considered the gold-standard to be either glucose or LBT, with different NAFLD association findings (16, 17). However, SIBO association with low LS and LF stages of liver diseases and, more significantly, with liver cirrhosis are consolidated data from the literature (53, 54). Third, the sample of this study did not include patients with NAFLD only. Thus, findings from the present study have to be considered as a pilot attempt to study the complex association between dysbiosis and liver diseases of different origin and stages vs. most recent reports focusing specifically either on NAFLD or on cirrhotics (18, 19). Fourth, we acknowledge that ultrasound assessment for LS grading has several limitations, as it is based on qualitative measurement and has low sensitivity for low-fat deposition in the liver, namely, lower than 20% of examined parenchyma (22, 23). Finally, data on histopathology are lacking in this study and cannot confirm a significant association between SIBO, endotoxemia levels, and NALFD/ALD LF stages observed in other reports (17, 18).

Further development of this study would be typing of fecal microbiota composition of patients. Indeed, SIBO just describes indirectly upper GI tract dysbiosis, and endotoxin is only a derived measurement of portal blood PAMPs. Thus, a deep study on deranged gut microbiota would guarantee a disease- and stage-specific real-time description of dysbiosis in these patients.

Therefore, larger prospective trials are warranted to confirm the preliminary results from this single-center exploratory prospective study.

## Data Availability Statement

The original contributions presented in the study are included in the article/supplementary materials, further inquiries can be directed to the corresponding author/s.

## Ethics Statement

The studies involving human participants were reviewed and approved by ASUR Marche Regional Ethical Committee. The patients/participants provided their written informed consent to participate in this study.

## Author Contributions

ES and LA contributed to conceptualization and the original idea of this manuscript. ER, VC, CR, and GS contributed to methodology and reviewed the literature. ES, LA, AS, and PS involved in validation and revised and validated the literature findings. ES, FL, and ER performed formal analysis. ES, MS, and FC contributed to investigation. ES, FL, and LA involved in data curation. ES, FL, LA, and GB did writing—original draft preparation. ES, LA, AS, and PS did writing—review and editing. ER, VC, and FC did visualization. ES, LA, AS, and CR involved in supervision. ES, LA, AS, and GB contributed to project administration. All authors contributed to the article and approved the submitted version.

## Conflict of Interest

The authors declare that the research was conducted in the absence of any commercial or financial relationships that could be construed as a potential conflict of interest.

## Publisher's Note

All claims expressed in this article are solely those of the authors and do not necessarily represent those of their affiliated organizations, or those of the publisher, the editors and the reviewers. Any product that may be evaluated in this article, or claim that may be made by its manufacturer, is not guaranteed or endorsed by the publisher.
